# A Journalistic War

**Published:** 1896-06

**Authors:** 


### A JOURNALISTIC WAR.

THE Ohio *Dental Journal* has been for some time endeavoring to correct the bad habit of a prominent trade journal for the offence of quoting without proper credit. This is such an old affair that we have long since ceased to notice it or to expect anything better.

Our objection to the course pursued by the *Items of Interest*—the journal especially condemned—is not so much a loose way of crediting, but the unjust method adopted in that journal of dividing an article into fractional parts and publishing as though these were distinct papers. This no editor is justified in doing, as it is a positive wrong done the author. If a journal must take its matter from other journals, let it be with justice to all concerned.

Trade has very devious methods, and it cannot be expected that the morality of any trade journal will rise any higher than the source of its power.

